# Association of Childhood Psychomotor Coordination With Survival Up to 6 Decades Later

**DOI:** 10.1001/jamanetworkopen.2020.4031

**Published:** 2020-04-30

**Authors:** G. David Batty, Ian J. Deary, Mark Hamer, Philipp Frank, David Bann

**Affiliations:** 1 Department of Epidemiology & Public Health, University College London, London, United Kingdom; 2 School of Biological & Population Health Sciences, Oregon State University, Corvallis; 3 Centre for Cognitive Ageing & Cognitive Epidemiology, University of Edinburgh, Edinburgh, United Kingdom; 4 Institute of Sport, Exercise & Health, University College London, London, United Kingdom; 5 Department of Behavioural Science and Health, University College London, London, United Kingdom; 6 Centre for Longitudinal Studies, University College London Institute of Education, London, United Kingdom

## Abstract

**Importance:**

Poorer performance on standard tests of motor coordination in children has emerging links with sedentary behavior, obesity, and functional capacity in later life. These observations are suggestive of an untested association of coordination with health outcomes, including mortality.

**Objective:**

To examine the association of performance on a series of psychomotor coordination tests in childhood with mortality up to 6 decades later.

**Design, Setting, and Participants:**

The British National Child Development Study (1958 Birth Cohort Study) is a prospective cohort study based on a nationally representative sample of births from England, Scotland, and Wales. A total of 17 415 individuals had their gross and fine motor psychomotor coordination assessed using 9 tests at ages 11 and 16 years. Data analysis for the present study was conducted from October 2016 to December 2019.

**Main Outcomes and Measures:**

All-cause mortality as ascertained from a vital status registry and survey records.

**Results:**

In this birth cohort study of 17 415 individuals who underwent a series of psychomotor coordination tests in childhood, follow up was conducted over several decades. Of the analytical sample of 12 678 individuals, 51% were male, and 72% came from a lower social group. Mortality surveillance between ages 12 and 58 years in an analytical sample of 17 062 men and women yielded 1072 deaths (766 661 person-years at risk). In survival analyses with adjustment for sex, higher scores on 7 of the 9 childhood coordination tests were associated with a lower risk of mortality in a stepwise manner. After controlling for early-life socioeconomic, health, cognitive, and developmental factors, lower mortality was statistically significantly associated with 3 tests: ball catching at age 11 years (0-8 vs 10 catches: hazard ratio [HR], 1.57; 95% CI, 1.19-2.07), match-picking at age 11 years (>50 vs 0-36 seconds: HR, 1.33; 95% CI, 1.09-1.63), and hopping at age 16 years (very unsteady vs very steady: HR, 1.28; 95% CI, 1.01-1.63).

**Conclusions and Relevance:**

The results of this cohort study suggest that childhood motor coordination is associated with lower mortality up to middle-age; these findings require replication.

## Key Points

**Question:**

Is performance on a series of psychomotor coordination tests in childhood associated with mortality up to 6 decades later?

**Findings:**

In this birth cohort study of 17 415 individuals who underwent a series of psychomotor coordination tests in childhood, follow up was conducted over several decades. After taking into account confounding factors, lower performance on 3 gross and fine motors skills tests in childhood was associated with elevated death rates up to 6 decades later.

**Meaning:**

The findings of this study suggest that childhood motor coordination is associated with lower mortality up to middle-age; replication in other contexts using complementary observational approaches is warranted.

## Teaser

This cohort study of data from the British National Child Development Study examines the association between children's performance on psychomotor tests and mortality at age 58 years.

## Introduction

Coordinated movement is the product of the dynamic and complex interplay of multiple neural mechanisms that are modulated by sensory inputs and spinal reflex loops.^1^ While there have been studies on the social and educational sequelae of poorer performance on standard tests that capture the capacity to coordinate movement,^2,3^ in parallel work, there is emerging evidence suggesting a detrimental association between poor coordinated movement and health-related outcomes in later life.

Thus, in extended follow-up of general population-based surveys into middle and older age, poorer motor skill scores in childhood that nonetheless lie within the normal range are associated with later physical inactivity,^4,5^ lower functional capacity,^6^ higher body mass index,^7^ mental health problems,^8,9^ and worse self-rated health.^9^ Findings from a national birth cohort study, for instance, suggest that higher scores on fine motor coordination tasks at age 15 years were associated with superior performance on physical function tests of standing balance and chair rising at age 53 years^6^—test results that themselves have been linked to greater life-expectancy.^10^ In a similar longitudinal study, lower scores on general motor function tests at age 11 years were associated with a higher risk of obesity 2 decades later.^7^ Linear associations were evident across the full range of coordination scores such that the association was not simply generated by children at the lowest end of the continuum in whom a diagnosis of coordination disorder was likely. In comparisons of the characteristics of children with such a diagnosis, however, there are also reports of a greater likelihood of increased levels of blood pressure and triglycerides, less-favorable cardiac output, and lower left-ventricular volume compared with unaffected controls.^11,12^

Taken together, these findings provide a prima facie case for the hypothesis that poorer scores on tests of psychomotor coordination in early life may be associated with mortality in adulthood. We tested this proposition using a 6-decade follow-up of over 17 000 members of the British National Child Development Study (1958 Birth Cohort Study).^13^ Given that children with lower coordination scores may come from disadvantaged social backgrounds and have less-favorable levels of cognitive function and birth characteristics—all of which are preadult risk factors for mortality in their own right^14,15^—we took these potential confounding factors into account, thus attempting to ascertain whether there was an independent association of mortality with earlier psychomotor coordination.

## Methods

Described in detail elsewhere,^13^ the National Child Development Study (or the 1958 British Birth Cohort Study) is an ongoing prospective birth cohort study initially comprising 17 415 births to women residing across Britain (England, Wales, and Scotland) in 1 week of 1958. Following data collection during the perinatal period, to date, there have been 10 follow-up surveys of study members in the National Child Development Study designed to monitor their physical, educational, social, and health development up to age 55 years.^13^ Responders remain broadly representative of the original sample.^16^ The present analysis of the data was conducted from October 2016 to December 2019. Ethical approval for data collection was granted by the South-East Multi-Centre Research Ethics Committee, and study participants provided informed consent. As is standard, ethical approval for statistical analyses of anonymized data from this birth cohort study was not required. This study followed the Strengthening the Reporting of Observational Studies in Epidemiology (STROBE) reporting guideline.^17^

### Assessment of Psychomotor Coordination

At age 7 years, qualitative judgments about the study member’s motor skills were sought from the teacher or parent, whereas at 11 and 16 years, psychomotor tests were administered by a school medical officer.^9^ Owing to the subjective nature of the enquiries at age 7 years, our main results are for the later tests, which included fine and gross motor skill evaluations.

When the children were aged 11 years, we used results from 6 tests of motor coordination. Children were asked to stand on their right foot for 15 seconds, to stand heel-to-toe for 15 seconds, and to walk backward for 20 steps along a line, hands on hips, and toe-to-heel. Performance on these tests was categorized into 1 of 3 groups (very steady, slightly unsteady, or very unsteady). Next, standing with their forearm horizontal, study members were instructed to bounce a ball, catching it with palm facing downward; the number of catches out of 10 attempts was recorded (categories: 0-8, 9, and 10 catches [best]). Participants were also asked to individually pick up and transfer 20 matches from one matchbox to another; the time taken was recorded and categorized into quartiles for the purposes of the present analyses (0-36 [fastest], 37-43, 44-50, and >50 seconds). In addition, on a printed grid of 200 squares, the children were asked to mark as many squares as possible within 1 minute; again, these scores were categorized into quartiles: (>126 [highest], 110-126, 95-109, and 0-94 squares). From the age 16 years survey, we used 3 coordination tests. The heel-toe standing balance and ball-catching tests were readministered together with a hopping test. In the latter test, participants jumped 1-legged between 4 lines spaced 2 feet apart, swiveled, then returned on the same leg. Performance was rated as very steady, slightly unsteady, and very unsteady.

These 9 coordination tests, among the earliest to be developed, were subsequently incorporated into batteries in common use today, including the Zurich Neuromotor Assessment,^18^ the Movement Assessment Battery for Children,^19^ and the McCarron Assessment of Neuromuscular Development.^20^ Interrater reliability and test-retest reliability of these test batteries are high, scores correlate moderately well with reaction time, and the results have diagnostic utility.^21^

### Potential Confounding Factors and Mortality

Birth weight was recorded by the midwives and gestational age was based on the number of days from the first day of the last menstrual period. At birth, enquiries were made about the occupational social class of the mother and father (6 categories, from professional to unskilled),^22^ maternal attendance in postcompulsory school, overcrowding in the home, maternal weight, and smoking status. When the study member was aged 7 years, mothers also responded to questions about whether the child had been breastfed and if they thought their child was right-handed, left-handed, or ambidextrous. Height and weight were measured by trained medical personnel at age 7 years using standardized protocols,^23^ and study members were administered the Southgate Group Reading Test and Problem Arithmetic Test.^24^

Pubertal timing was ascertained at age 16 years using sex-specific methods.^25^ In boys, trained medical personnel visually rated pubic hair development as absent, sparse, intermediate, or adult. In girls, age of menarche was recalled by the student (if missing, a parent report was used). We regarded pubertal development as being advanced if boys had adult hair development or if the girls’ age at menarche was 11 years or younger. The presence of multiple health conditions at age 7 years was ascertained by medical officers in schools and by parental interview in the home. These conditions were grouped into 12 classifications, for example, asthma/bronchitis, allergies, chronic medical conditions, and chronic physical/mental handicap injuries.^26^ Common infectious illnesses were not taken into account as these were reported by almost all the study sample. Frequency of participation in indoor and outdoor sport and games, was also assessed at age 16 years (often, sometimes, hardly ever, no chance).

Vital status was ascertained up to age 58 years (December 2016), using death certificates supplied by the Office for National Statistics^27^ and/or notifications given during fieldwork following direct contact with participants’ family members.^28^ All-cause mortality from the date of coordination test administration (ages 11 or 16 years) to age 58 years was used in analyses.

### Statistical Analysis

To provide some insights into the confounding structure herein, associations of covariates with coordination were examined (χ^2^ tests), as were associations of potential confounders with mortality risk (Cox proportional hazards regression models^29^). In the main analyses, the proportional hazard assumption was tested by both visually inspecting hazard estimates and by testing Schoenfeld residuals^30^; there was no evidence for violation. We then summarized the link between each test of coordination and mortality using Cox models. Hazard ratios (HRs) with accompanying 95% CIs were computed and initially adjusted for sex (no age adjustment was required given that study members are aged within 7 days of one another), followed by potential confounding variables. To address the potential for reverse causality due to injury or illness, we incorporated a 1-year lag interval was used between exposure ascertainment and mortality ascertainment. To reduce any possible effect of missing data on statistical power and the occurrence of selection bias,^31^ missing exposure and confounder data were estimated using multiple imputation with 10 such data sets. Briefly, chained equations were developed to estimate missing values, using either logistic or linear regression models depending on the measurement. These imputed data sets were then used to estimate associations between coordination and mortality, accounting for uncertainty in the exact value of any missing data. Survival analyses based on the imputed data set revealed results similar to those apparent based on the nonmissing data set. Statistical significance was set at 2-sided *P* < .05. Statistical analysis was conducted using Stata, version 15 (StataCorp LP).

## Results

In this birth cohort study of 17 415 individuals who underwent a series of psychomotor coordination tests in childhood, follow up was conducted over several decades. Of the analytical sample of 12 678 individuals, 51% were male, and 72% came from a lower social group. In general, coordination test results at both ages 11 and 16 years were heavily skewed to the left, such that high scores were commonplace. We first ascertained whether psychomotor coordination test scores at ages 11 and 16 years were linked to study covariates. For example, ball-catching test results at age 11 years were associated with cognitive ability, laterality, and health (Table), such that children with lower coordination scores performed less favorably in these domains; however, for most of the potential confounding factors there was no evidence of a link. A somewhat dissimilar pattern of association was evident at age 16 years, with a higher prevalence of socioeconomic disadvantage (parental manual social class and household overcrowding), an absence of breastfeeding, and increased maternal weight and study members’ weight was apparent in children who were less successful on the hopping test (eTable in the Supplement).

**Table.** Study Member Characteristics According to Ball-Catch Test Results at Age 11 Years

Characteristic	Age at measurement, y	No.	No. of ball catches, No. (%)			*P* value for heterogeneity^a^
			10 (best)	9	8-0	
Sex						
Male	0	12 583	5068 (49.7)	845 (56.1)	533 (60.4)	<.001
Manual parental occupational social class	0	12 547	7235 (71.2)	1090 (72.6)	650 (73.8)	.36
Mother did not attend school post compulsory level	0	11 948	7153 (74.0)	1044 (72.7)	650 (77.3)	.16
Overcrowding, >2 persons/room	0	11 672	2896 (30.7)	428 (30.5)	251 (30.5)	.94
Mother smoked prior to pregnancy	0	11 640	3676 (39.0)	518 (37.1)	303 (37.1)	.36
Mother did not breastfeed	7	11 257	2787 (30.5)	415 (31.3)	270 (34.6)	.11
Mother’s weight, >76.2 kg	0	11 727	1171 (12.3)	161 (11.4)	86 (10.5)	.73
Participants’ weight, >27.2 kg	7	10 778	1179 (13.5)	158 (12.4)	96 (12.7)	.54
Participants’ height, <1.14 m	7	10 966	479 (5.4)	90 (7.0)	71 (9.4)	<.001
Birth weight, <2.5 kg	0	11 601	519 (5.5)	81 (5.8)	59 (7.2)	.13
Gestational age <9 mo	0	10 814	1899 (21.6)	254 (20.0)	160 (21.1)	.42
Cognition						
Low reading test score	7	11 423	1008 (10.9)	199 (14.6)	165 (20.8)	<.001
Low math test score	7	11 389	2409 (26.1)	400 (29.6)	306 (38.4)	<.001
Pubertal timing, advanced	16	8170	2258 (34.2)	349 (35.6)	206 (34.8)	.97
Laterality, left or mixed handed	7	11 336	820 (8.9)	195 (14.6)	147 (18.6)	<.001
Health^b^						
Chronic medical condition	7	10 962	874 (9.8)	138 (10.7)	116 (15.3)	<.001
Chronic physical/mental handicap	7	10 968	556 (6.2)	71 (5.5)	51 (6.8)	.48

^a^ *P* values derived from χ^2^ tests (overall heterogeneity); *P* values calculated using all available categories. Number of people refers to those with data on the characteristic, ball catch test results, and mortality.

^b^ Two of the 12 health conditions are reported for the purposes of illustration. In regression analyses, all 12 factors were taken into account.

**Figure 1.**
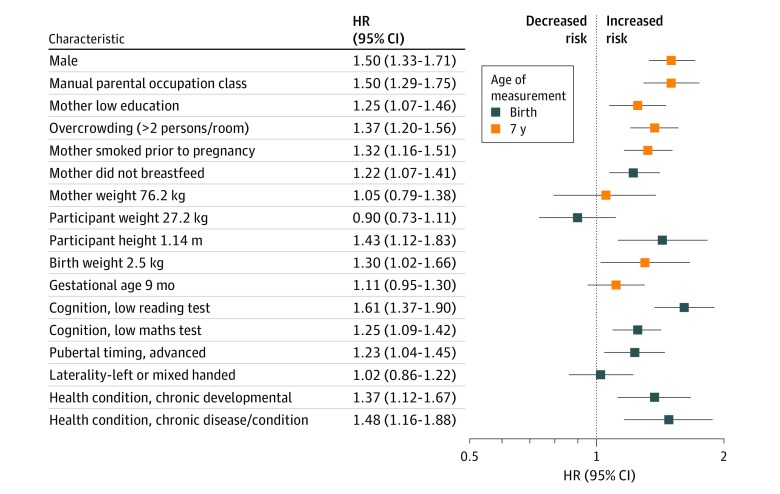
Association of Early Life Characteristics With Total Mortality Up to Age 58 Years Unadjusted analysis shown. HR indicates hazard ratio; error bars, 95% CI.

Mortality surveillance between ages 12 and 58 years in a maximum analytical sample of 17 062 men and women yielded 1072 deaths (766 661 person-years at risk). With the exception of the mother’s weight, study member’s weight in childhood, gestational age, and laterality, the remaining 13 covariates were related to higher mortality rates in the expected directions in univariate analyses (Figure 1). For instance, associations with death were evident for manual social class (HR, 1.50; 95% CI, 1.29-1.75), poor reading test performance (HR, 1.61; 95% CI, 1.37-1.90), low birth weight (HR, 1.30; 95% CI, 1.02-1.66), and suboptimal physical growth (HR, 1.43; 95% CI, 1.12-1.83).

**Figure 2.**
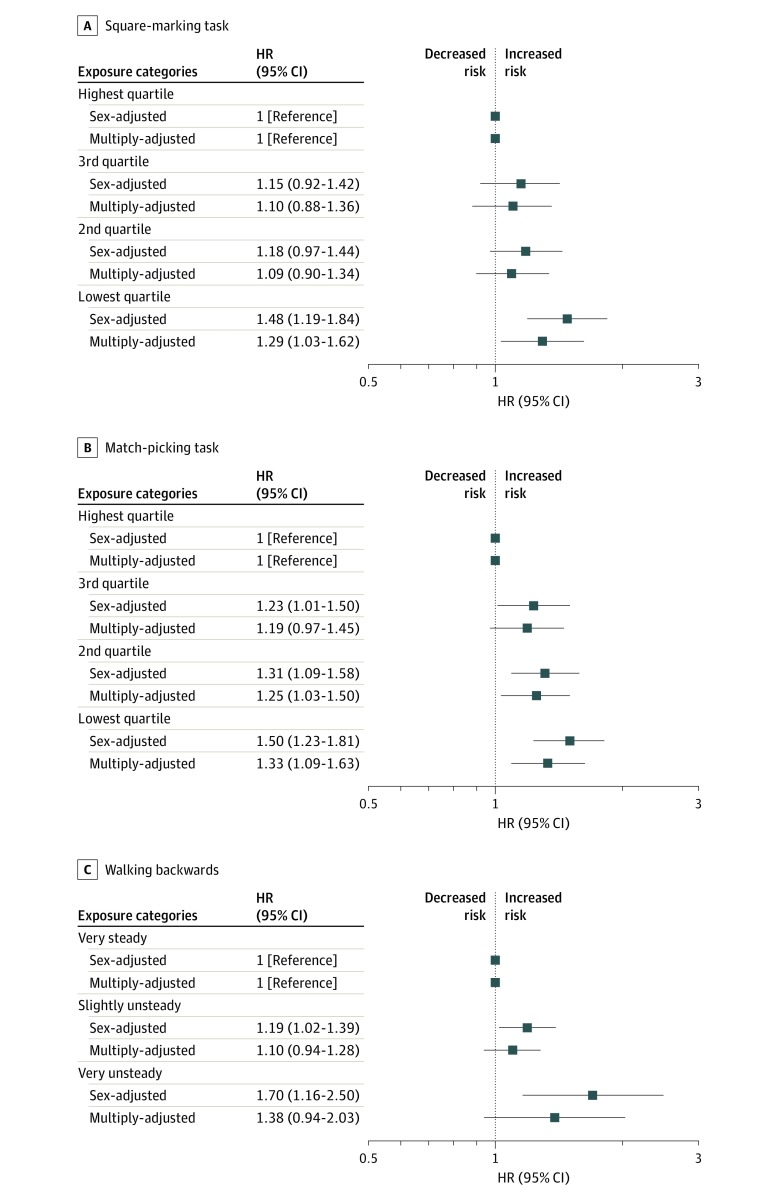
Association of Square Marking, Match Picking, and Walking Backward at Age 11 Years With All-cause Mortality by 58 Years Tests were conducted for square marking (A), match picking (B), and walking backward (C). HR indicates hazard ratio; error bars, 95% CI. Multiple adjustment is adjustment for childhood socioeconomic, health, cognitive, and developmental factors as listed in Table 1.

**Figure 3.**
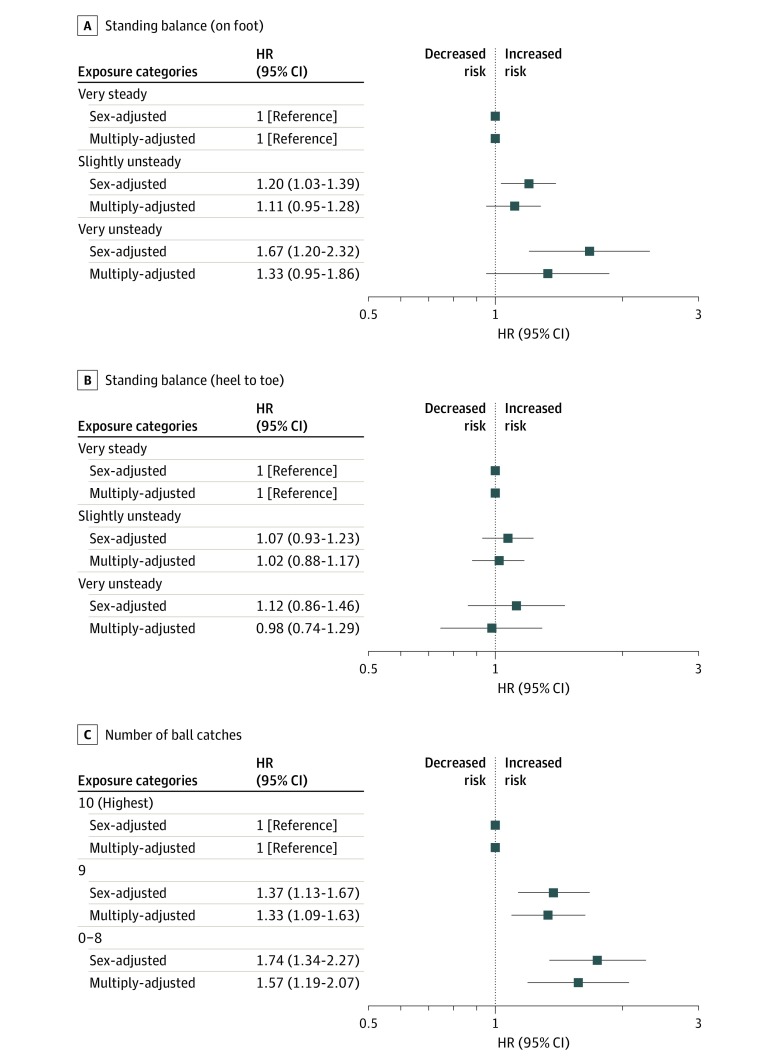
Association of Standing Balance (Foot and Heal-to-Toe) and Ball-Catching Tests at Age 11 Years With All-cause Mortality by 58 Years Tests were conducted for standing balance on foot (A), standing balance heel to toe (B), and number of ball catches (C). HR indicates hazard ratio; error bars, 95% CI. Multiple adjustment is adjustment for childhood socioeconomic, health, cognitive, and developmental factors as listed in Table 1.

**Figure 4.**
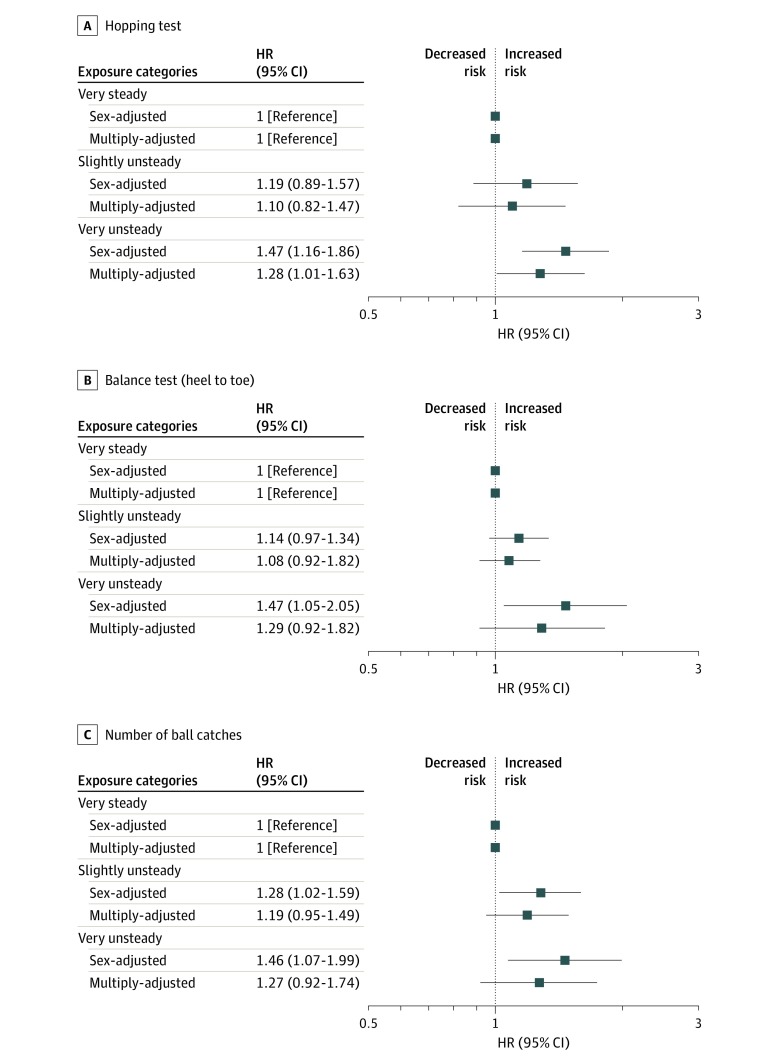
Association of Standing Balance (Foot and Heal-to-Toe) and Ball-Catching Tests at Age 16 years With All-cause Mortality by 58 Years Tests were conducted for standing balance on foot (A), standing balance heel to toe (B), and number of ball catches (C). HR indicates hazard ratio; error bars, 95% CI. Fully adjusted is adjustment for childhood socioeconomic, health, cognitive, and developmental factors as listed in Table 1.

In Figure 2 and Figure 3, for tests administered at age 11 years, and in Figure 4, for tests at age 16 years, the associations with mortality while controlling for potentially confounding variables that might account for all or part of the gradient are shown. In analyses with sex in the statistical model, most test scores from both time points—7 of the 9 included—were inversely associated with mortality risk, such that a higher concentration of deaths was apparent in children who had lower coordination scores. For example, at age 11 years, the lowest-performing group on the 1-foot standing balance challenge had a 67% greater risk of death than the highest-scoring category (very unsteady vs very steady: HR, 1.67; 95% CI, 1.20-2.32). At age 16 years, using the same categorizations, suboptimal results on the hopping test were associated with nearly a 50% increase in mortality rate (HR, 1.47; 95% CI, 1.16-1.86). Where positive results were apparent, associations were typically stepwise across the full range of coordination scores.

In analyses with 16 covariates added to the multivariable model, there was clear attenuation across all associations, and statistically significant associations were evident for only 3 tests: ball catching at age 11 years (0-8 vs 10 catches: HR, 1.57; 95% CI, 1.19-2.07), match-picking at age 11 years (>50 vs 0-36 seconds: HR, 1.33; 95% CI, 1.09-1.63), and hopping at age 16 years (very unsteady vs very steady: HR, 1.28; 95% CI, 1.01-1.63). The strongest association with mortality was evident with the parent or teacher’s subjective reporting of study member’s coordination at 7 years as opposed to the results from the tests administered at later ages. After multiple adjustments, poor teacher-assessed hand coordination (vs not poor) was related to a 64% increased risk of mortality (HR, 1.64; 95% CI, 1.28-2.10), and a poor overall rating producing similar results (HR, 1.70; 95% CI, 1.27-2.28) (eFigure in the Supplement).

## Discussion

The main findings of the present study were that better performance on most childhood coordination tests was associated with a lower risk of mortality up to 6 decades after measurement. Most associations were graded across the test score categories. After further control for an array of socioeconomic, health, cognitive, and developmental factors, however, these associations held at conventional levels of statistical significance only for the ball-catching, match-picking duration, and hopping tests. We were able to recapitulate established associations from other life-course–orientated studies whereby the early life characteristics of socioeconomic position,^32,33^ cognitive function,^15,34,35^ birth characteristics,^14^ and postnatal growth^36,37^ were all linked to the risk of death up to 6 decades later (Figure 1). These findings increase confidence in our results for psychomotor coordination.

The strongest association with mortality was evident with the parent or teacher’s subjective reporting of study member’s coordination at 7 years (eFigure in the Supplement) as opposed to the results from the tests administered at later ages. These differences may be because the evaluations were more prone to the observer’s knowledge of existing psychomotor problems in the school children, including those with a developmental coordination disorder.

### Mechanisms of the Associations

The robustness to statistical adjustment of selected gradients in coordination and death raised the possibility of mechanistic links that may be direct or indirect. Our outcome in these analyses was mortality from all causes and, although we did not have data on individual causes of death, in higher-income countries, mortality by age 60 years is known to broadly comprise chronic disease (chiefly cardiovascular disease and cancer) and external causes of death (chiefly suicide and accidents, particularly road traffic). We speculate that the association between childhood coordination and external cause of death, such as road traffic accidents, may be direct: superior coordination results may capture an ability to rapidly respond to threat by removing oneself from high-risk situations, whether in the position of pedestrian or vehicle driver, so minimizing injury and preserving life.^38^ A further direct association of coordination with mortality may be via system integrity, which may have more relevance to chronic disease. First posited in the context of intelligence,^9^ system integrity concerns the suggestion, as yet not widely explored, that scoring well on tests of psychomotor coordination might be an indicator of a more general tendency for complex systems to function optimally. Thus, children with higher test scores may also have superior functioning of vital organs, such as the heart, lung, liver, and kidneys and, as a result, enhanced life expectancy.^39,40^

Other associations between coordination and chronic disease may be indirect. That is, these relationships might be at least partially mediated by characteristics known to be affected by coordination that are also established risk factors for disease. One such factor might be adult mental health problems, themselves linked to earlier measurement of coordination,^9^ which have been linked to mortality risk.^41,42,43^ Although we were interested in the association, if any, of normal range coordination scores on mortality risk, as described, there is a suggestion that, relative to unaffected controls, children with developmental coordination disorder experience raised levels of blood pressure and triglycerides, less-favorable cardiac output, and lower left-ventricular volume.^11,12^ These mechanisms may also be implicated in children who scored suboptimally on the coordination test, but who nonetheless did not meet the criteria for a diagnosis of developmental coordination disorder. Although some of these intermediary data were collected in middle-aged participants in the present study, the amount of data on the number of deaths in those with both childhood coordination and adult risk factors is currently too low to facilitate reliable mediation analyses. In addition, it is plausible that there is a genetic association between the known genetic variants linked to longevity^44^ and those that predispose participants to better coordination. Should a large-scale, genome-wide association study of life expectancy be performed, this issue could be investigated using statistical genetic techniques.

To our knowledge, this is the first study to test the hypothesis that the results of early-life tests of coordination are associated with survival into adulthood, so direct comparison with extant results is not possible. As described, however, there may be some evidence that poorer motor skill scores in childhood are linked to mental health problems^8,9^ and worse self-rated health.^9^ Several of the tests of coordination conducted in childhood in the present study appear to bear some useful resemblance to standard assessments of physical capacity used in populations of older adults.^45^ These assessments include the 1-leg and heel-to-toe balance tests and the walking test; poorer performance on these measures is associated with earlier mortality with similar magnitude to those in the present analyses.^10^ In these older populations, reduced performance on such tests is likely to be at least partially ascribed to a higher occurrence of comorbidity (eg, hypertension, stroke, and cognitive impairment). By using a cohort whose members had their coordination tested in childhood when such illnesses would generally be rare at the time of assessment, we may have at least partially circumvented this issue of reverse causality.

### Strengths and Limitations

While our study has some strengths, including its novelty, its array of early-life confounding factors, and our focus on coordination tests that are widely used today, it has limitations. First, to maintain sufficient numbers of deaths to facilitate analyses, we were not able to use potential mediating variables collected in later life. Second, although the use of coordination data from several decades ago is necessary given the research question, this lengthy interval nonetheless raises the issue of whether contemporaneously gathered data would reveal the same patterns of association with mortality. If the association is causal, which is currently moot, then the era of data collection is irrelevant. Third, given the large number and array of covariates included in our statistical models, particularly in socioeconomic, cognitive, and health domains, it is possible that the reported HRs are underestimates of the risk of lower levels of coordination. This underestimation may have occurred because several of the covariates used might capture the same characteristic. For instance, parental social class, mother’s educational level, and home overcrowding are all indicators of socioeconomic status that many investigators use individually. Similarly, coordination and cognition could be quantifying the same unmeasured neurobiologic factor that ultimately determines mortality risk. In addition, having necessarily conducted multiple tests in the course of our analyses, some positive results could have surfaced by chance.

## Conclusions

In this cohort study, selected fine and gross motor skills test results appear to be associated with mortality risk up to 6 decades later. These findings require replication.

## Notes

Supplement. **eTable.** Study Member Characteristics According to Hopping Test Score at Age 16 Years **eFigure.** Multiply-Adjusted Hazard Ratios (95 Confidence Intervals) for the Relation of Qualitative Psychomotor Coordination Evaluation at Age 7 Years With All-cause Mortality by 58 Years
