# Supplementary material for: Association of Childhood Psychomotor Coordination With Survival Up to 6 Decades Later
Source: JAMA Netw Open. 2020 Apr 30;3(4):e204031. doi: 10.1001/jamanetworkopen.2020.4031 (PMC7193332; doi:10.1001/jamanetworkopen.2020.4031)
Supplement: Supplement. — eTable. Study Member Characteristics According to Hopping Test Score at Age 16 Years eFigure. Multiply-Adjusted Hazard Ratios (95 Confidence Intervals) for the Relation of Qualitative Psychomotor Coordination Evaluation at Age 7 Years With All-cause Mortality by 58 Years [file jamanetwopen-3-e204031-s001.pdf]

Supplementary Online Content

Batty GD, Deary IJ, Hamer M, Frank P, Bann D. Association of childhood psychomotor coordination with survival up to 6 decades later. *JAMA Netw Open*. 2020;3(4):e204031. doi:10.1001/jamanetworkopen.2020.4031

eTable. Study Member Characteristics According to Hopping Test Score at Age 16 Years

eFigure. Multiply-Adjusted Hazard Ratios (95% Confidence Intervals) for the Relation of Scores from a Qualitative Psychomotor Coordination Evaluation at Age 7 Years With All-cause Mortality by 58 Years

This supplementary material has been provided by the authors to give readers additional information about their work.

© 2020 Batty GD et al. *JAMA Network Open.*

eTable. Study member characteristics according to hopping test score at age 16 years

Characteristic (age measured)		Hopping test score (N, %)			
	N	Very steady	Slightly unsteady	Slightly-very unsteady	P-value for heterogeneity
Male	10946	4745 (51.9)	354 (46.9)	529 (50.1)	0.02
Manual parental occ. class (0 years)	10641	6256 (70.4)	536 (73.6)	765 (74.8)	0.01
Mother did not attend post compulsory school (0 years)	10170	6218 (73.1)	504 (73.1)	737 (75.8)	0.3
Overcrowding (>2persons/room; 0 years)	9941	2469 (29.7)	217 (32.1)	309 (32.4)	0.01
Mother smoked prior to pregnancy (0 years)	9913	3154 (38.0)	261 (39.2)	383 (40.2)	0.7
Mother did not breastfeed (7 years)	9439	2323 (29.4)	228 (35.7)	313 (34.9)	<0.001
Mother's weight (0 years) >76.2 kg	9962	953 (11.4)	99 (14.7)	145 (15.3)	<0.001
Participants' weight >27.2 kg (7 years)	9027	959 (12.7)	109 (17.7)	138 (16.3)	<0.001
Participants' height <1.14 m (7 years)	9198	430 (5.6)	40 (6.4)	54 (6.2)	0.6
Birth weight <2.5kg (0 years)	9891	442 (5.3)	40 (6.1)	66 (7.0)	0.1
Gestational age <9 months (0 years)	9196	1592 (20.6)	128 (20.9)	197 (22.6)	0.4
Cognition, low reading test (7 years)	9609	841 (10.5)	83 (12.9)	169 (18.3)	<0.001
Cognition, low maths test (7 years)	9578	2049 (25.6)	211 (32.8)	333 (36.2)	<0.001
Pubertal timing, advanced (7 years)	9874	2912 (35.1)	215 (32.5)	295 (31.9)	0.3
Laterality – left or mixed handed (7 years)	9511	821 (10.3)	70 (10.8)	101 (11.2)	0.5
Health condition, chronic developmental (7 years)	9179	717 (9.3)	72 (11.5)	145 (16.6)	<0.001
Health condition, chronic disease/condition (7 years)	9196	451 (5.9)	45 (7.2)	66 (7.5)	0.08

Number of people refers to those with data on the characteristic, ball catch test results, and mortality.

© 2020 Batty GD et al. *JAMA Network Open.*

eFigure. Multiply-adjusted hazard ratios (95% confidence intervals) for the relation of scores from a qualitative psychomotor coordination evaluation at age 7 years with all-cause mortality by 58 years

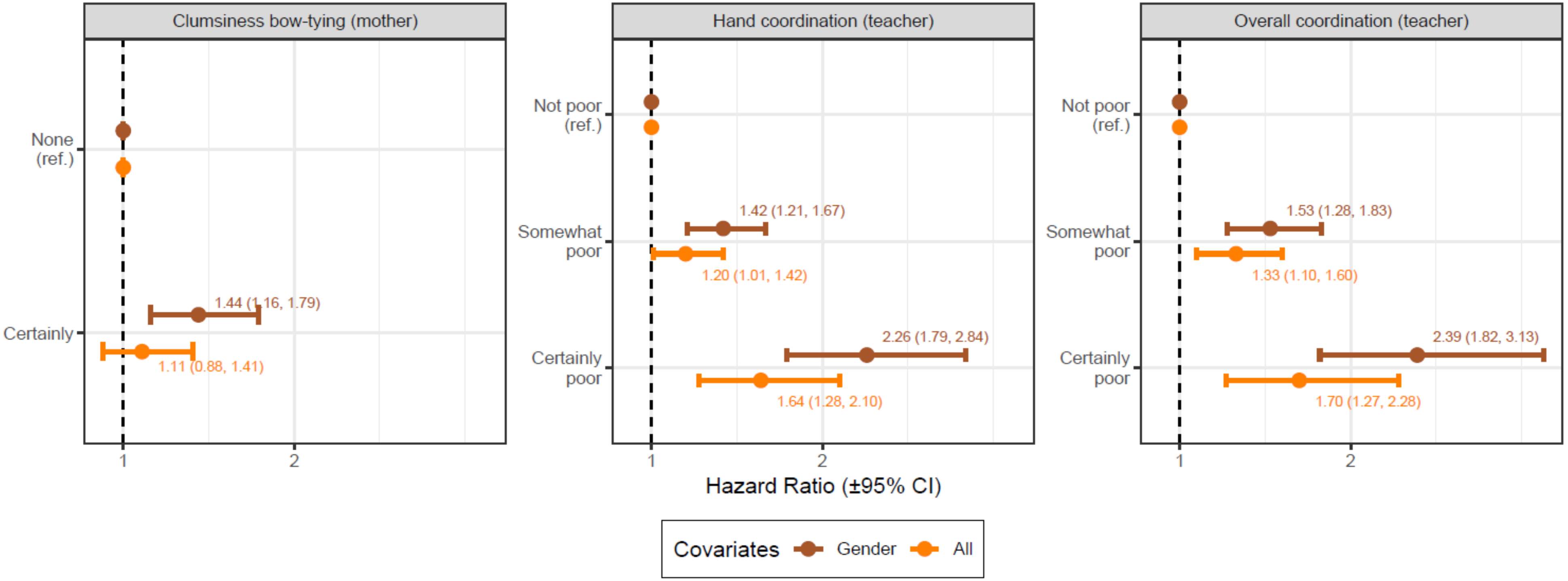

All covariates comprise childhood socioeconomic, health, cognitive, and developmental factors as listed in table 1. At age 7 years, mothers and teachers responded to three enquiries regarding the coordination of the study member. Mothers were asked if the child displayed awkwardness or clumsiness when typing a bow which had a binary response (‘none’, ‘certainly’). Teachers were asked whether the participant had poor hand and poor general coordination (response categories: ‘doesn’t apply’, ‘applies somewhat’, ‘certainly applies’).

© 2020 Batty GD et al. *JAMA Network Open.*
